# Decapeptide Inducer Promotes the Conidiation of Phytopathogenic *Magnaporthe oryzae* via the Mps1 MAPK Signaling Pathway

**DOI:** 10.3390/ijms26125880

**Published:** 2025-06-19

**Authors:** Mengya Yang, Yanan Liu, Jianhua Qi

**Affiliations:** College of Pharmaceutical Science, Zhejiang University, Yu Hang Tang Road 866, Hangzhou 310058, China; 22219083@zju.edu.cn (M.Y.); liuyanan1231@zju.edu.cn (Y.L.)

**Keywords:** *Magnaporthe oryzae*, MCIDP, conidiation, Mps1 MAPK signaling pathway

## Abstract

*Magnaporthe oryzae* (*M. oryzae*) is a phytopathogenic fungus that inflicts damage on vital crops, particularly rice. Its asexual reproduction leads to the generation of numerous conidia, which is a critical factor contributing to the prevalence of rice blast disease. However, the molecules regulating the asexual reproduction of *M. oryzae* are unknown. In our study, to identify the molecules capable of regulating the asexual reproduction of *M. oryzae*, compositions of the complete medium (CM) were screened. Results showed that acid-hydrolyzed casein (AHC) could remarkably promote conidial production. One *M. oryzae* conidiation inducer was isolated from AHC using high-performance liquid chromatography (HPLC) under the guidance of bioassay. Its structure was further elucidated as a decapeptide compound (pyroGlu-EQNQEQPIR) by LC-MS/MS, chemical synthesis, and conidium-inducing assays, named *M. oryzae* conidiation inducer decapeptide (MCIDP). MCIDP could significantly promote the conidiation of *M. oryzae* and two other filamentous ascomycetes (*Botrytis cinerea* and *Fusarium graminearum*). The Mps1 MAPK cascade signaling pathway is crucial for conidiation, and the effect of MCIDP on this pathway was investigated to elucidate the mechanism underlying conidiation enhancement. qRT-PCR analysis demonstrated that MCIDP could remarkably upregulate the gene expression within the Mps1 MAPK cascade signaling pathway, especially the *WSC2*, *WSC3*, *PKC1*, *MKK1*, *MPS1,* and *MIG1*. Furthermore, the Δ*Mowsc1*, Δ*Mowsc2*, Δ*Mowsc3*, and Δ*Momid2* mutant strains were constructed. Bioassay results showed that MCIDP failed to promote conidial formation and hyphal growth in these mutant strains. These findings indicate that MCIDP promotes conidiation of *M. oryzae* by modulating the Mps1 MAPK signaling pathway.

## 1. Introduction

Approximately half of the world’s population relies on rice as a primary food source. Rice blast is one of the most destructive diseases affecting global rice production. Its pathogen, *Magnaporthe oryzae* (*M. oryzae*), is a filamentous ascomycete [1] and encompasses both asexual and sexual reproductive phases in the life cycle [2]. This fungus predominantly completes its life cycle and disease cycle through asexual reproduction, which leads to the devastating rice blast disease affecting rice crops worldwide [3,4]. Rice blast disease tends to erupt during the maturation phase of rice, which makes it a significant menace to rice cultivation, leading to output declines ranging from 10% to 30% in average years and up to a drastic 50% decrease in more severe cases [5]. Recognized as one of the top 10 plant pathogens, *M. oryzae* can also infect other cereal crops, such as barley, wheat, and millet [6,7], threatening food security and causing significant economic losses.

Asexual and sexual reproduction are crucial biological events in the life cycle of microorganisms. Asexual reproduction in nature often leads to the rapid spread of diseases [8]. Discovering the endogenous signaling molecules of asexual and sexual reproduction and their specific receptors contribute to the discovery of specific antagonists and provide a theoretical basis and potential targets for new antimicrobial agents. In previous studies, we focused on finding endogenous signaling molecules that initiated sexual reproduction in *Phytophthora* and successfully identified the hormones α1 and α2 [9,10,11]. Additionally, we identified the hormone FARI (*Fusarium* asexual reproduction inducer) that regulated its asexual reproduction in *Fusarium graminearum* (*F. graminearum*) [12]. However, the signaling molecules regulating the asexual development and conidiation of *M. oryzae* have not been reported. To confirm whether the asexual reproduction of fungi is generally regulated by hormones, we aim to determine the endogenous hormones regulating the asexual reproduction of *M. oryzae*.

The establishment of a conidium-inducing assay method is challenging because fungal endogenous hormones are only available in trace amounts [12] and can be produced by *M. oryzae* itself when conducting a bioassay. Finding a positive control is necessary to improve the stability of the bioassay system. *M. oryzae* laboratory strains derived from Guy11 have been successfully grown on the complete medium (CM) for many years [13]. Given that the lycosides A−D isolated from vegetable juice could inhibit the asexual reproduction of *Phytophthora* [14], the compositions of CM were screened, and it was found that acid-hydrolyzed casein (AHC) significantly promoted the conidiation of *M. oryzae*. In the present study, we focused on the inducers of *M. oryzae* asexual reproduction from AHC and the related mechanisms.

The conidial formation of phytopathogenic fungi is regulated by multiple signaling pathways. Dean et al. reported that the mitogen-activated protein kinase (MAPK) signaling pathway is involved in regulating the asexual reproduction of *M. oryzae* [2]. It has five branches, among which the signal transduction of the Mps1 pathway is crucial for conidial formation [2,15,16]. Its upstream sensors, Wsc family proteins (Wsc1/2/3) and Mid2, have relatively conserved regulatory effects on fungal asexual reproduction and cell wall integrity [17,18].

The core genes in the Mps1 MAPK pathway are conserved and play an important regulatory role in conidial formation. *PKC1* is located upstream of this pathway, and its downregulation is accompanied by significant impairment in fungal growth and a reduction in conidia formation [19]*. MCK1* is crucial for the conidial formation of *M. oryzae*, and the conidiation of Δ*Momck1* is significantly impaired [20]. Meanwhile, the Δ*Momkk1* mutant almost does not produce conidia [21]. Some researchers found that the Δ*Momps1* mutant exhibits significantly reduced aerogenous mycelium growth and conidia [22]. In addition, transcription factors regulated by the Mps1 MAPK cascade include Mig1, Swi6, and Gti1, the latter of which is a regulator of many effector genes [16]. Among them, Δ*Momig1* exhibits slightly reduced aerial hyphal growth and conidiation compared with the wild-type [23]. The Δ*Mogti1* mutant has approximately 95% less conidial formation than the wild-type and complementary strains, and the conidia are also abnormal in appearance [24]. Approximately 40% of the conidia produced by the Δ*Moswi6* mutant are abnormal and have only one septum [25].

In conclusion, this study reported the isolation and characterization of a *M. oryzae* conidiation inducer decapeptide (MCIDP) from the AHC and its mechanism of action for conidial formation.

## 2. Results

### 2.1. Acid-Hydrolyzed Casein Significantly Promotes M. Oryzae Conidial Production

**Figure 1:**
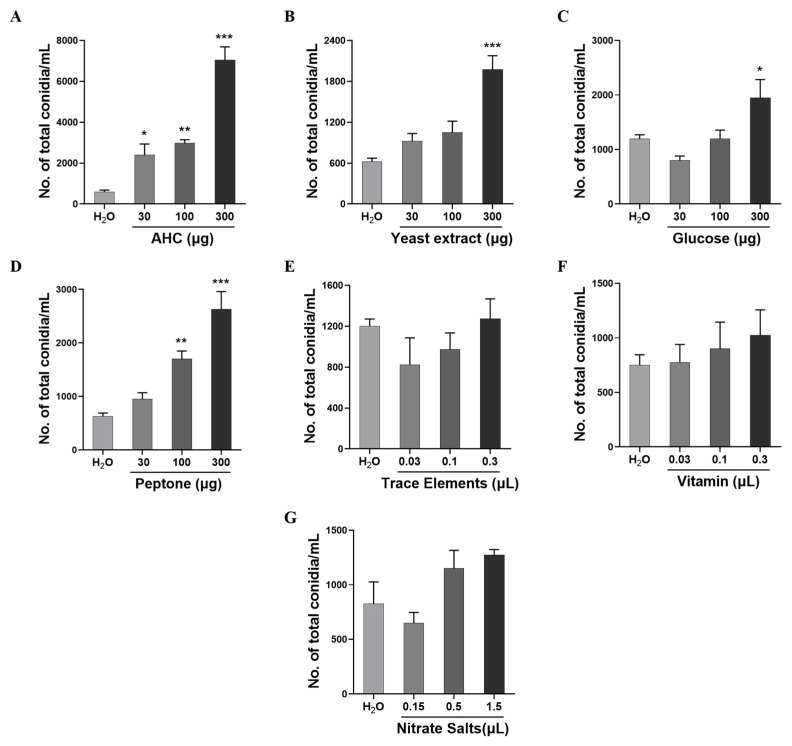
The impact of AHC, yeast extract, glucose, peptone, TE, vitamins, and nitrate salts on the production of *M. oryzae* conidium. The effects of 30, 100, and 300 μg/mL of AHC (**A**), yeast extract (**B**), glucose (**C**) and peptone (**D**), 0.03, 0.1, and 0.3 μL 1000 × trace elements solution (**E**), and 1000 × vitamin solution (**F**), 0.15, 0.5 and 1.5 μL 200 × solution (**G**) on the conidia formation of the *M. oryzae* strain Guy11 were tested on the 24-well plates; the negative control is sterilized water. The experiment was repeated three times. The data were expressed as a mean ± SEM. *, **, and *** represent a significant difference as compared to the negative group at *p* < 0.05, 0.01, and 0.001, respectively.

Complete medium (CM) is a commonly used medium for *M. oryzae* laboratory culture and can efficiently support growth and conidiation [13]. In the process of searching for positive control to improve the stability of the bioassay system, we screened the activity of CM medium compositions using the 24-well plate liquid culture bioassay method (Figure 1). Our findings indicated that AHC at concentrations of 30, 100, and 300 μg/mL (Figure 1A), yeast extract at 300 μg/mL (Figure 1B), glucose at 300 μg (Figure 1C), and peptone at 100 and 300 μg (Figure 1D) significantly stimulated conidial production. Among these factors, AHC exhibited the most pronounced effect on conidial yield (Figure 1A). Meanwhile, treatment with trace elements, vitamins, and nitrate salts induced no significant change in conidial formation (Figure 1E–G).

### 2.2. Decapeptide MCIDP from Acid-Hydrolyzed Casein Significantly Promotes Conidiation

**Figure 2:**
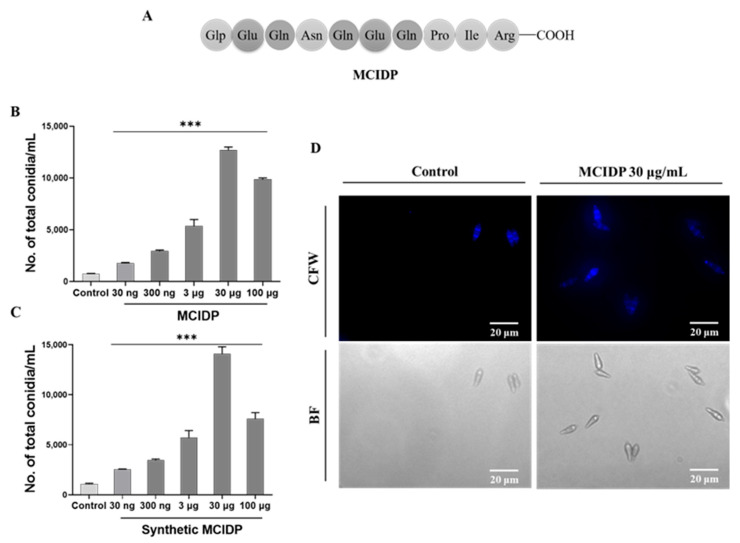
Chemical and biological characteristics of MCIDP. (**A**) The amino sequence of MCIDP. (**B–C**) The effects of 30 ng, 300 ng, 3 μg, 30 μg, and 100 μg/mL of isolated MCIDP (**B**) or synthetic MCIDP (**C**) on the production of conidia by the *M. oryzae* strain Guy11 were tested; the negative control was sterilized water. The experiment was repeated three times. The data were expressed as a mean ± SEM. *** represents a significant difference as compared to the negative group at *p* < 0.001. (**D**) Micrographs of conidia induced by 30 μg/mL of MCIDP in *M. oryzae*; (**up**): fluorescence micrograph; (**down**): photomicrograph. The fluorescence image of conidia stained with calcofluor white (CFW) was acquired with an Olympus IX53 inverted fluorescence microscope. Conidia were obtained after the filtration of hyphae and agar. BF: bright field. Scale bar: 20 μm.

AHC could significantly promote conidiation. In this study, under the guidance of bioassay, a decapeptide with significant conidium-inducing activity was isolated and purified from AHC, designated as MCIDP. The amino sequence of MCIDP is displayed in Figure 2A. Initially, we observed the conidial formation-promoting activity of MCIDP. The *M. oryzae* strain Guy11 was treated with different doses of MCIDP (30 ng/mL, 300 ng/mL, 3 μg/mL, 30 μg/mL, and 100 μg/mL), and sterilized water was used as the negative control. The results showed that MCIDP significantly induced conidiation at a dose of 30 ng/mL. The number of total conidia increased in a dose-dependently increased in the range of 30 ng/mL–30 μg/mL, peaked at 30 μg/mL, and slightly decreased at 100 μg/mL (Figure 2B). To further confirm its structure, we synthesized MCIDP according to the sequenced pyroGlu-EQNQEQPIR and assayed its activity. The activity of synthetic MCIDP was similar to that of the isolated one (Figure 2C). This finding demonstrated that MCIDP is a decapeptide structure that has a sequence of pyroGlu-EQNQEQPIR and can significantly promote the conidiation in Guy11. Additionally, Figure 2D shows the photomicrograph of the conidia obtained in the conidium-inducing assay without or with 30 μg/mL MCIDP, indicating that MCIDP could significantly induce conidial production but did not affect conidial morphology.

### 2.3. MCIDP Significantly Induced Conidial Formation in Two Other Filamentous Phytopathogenic Ascomycetes

**Figure 3:**
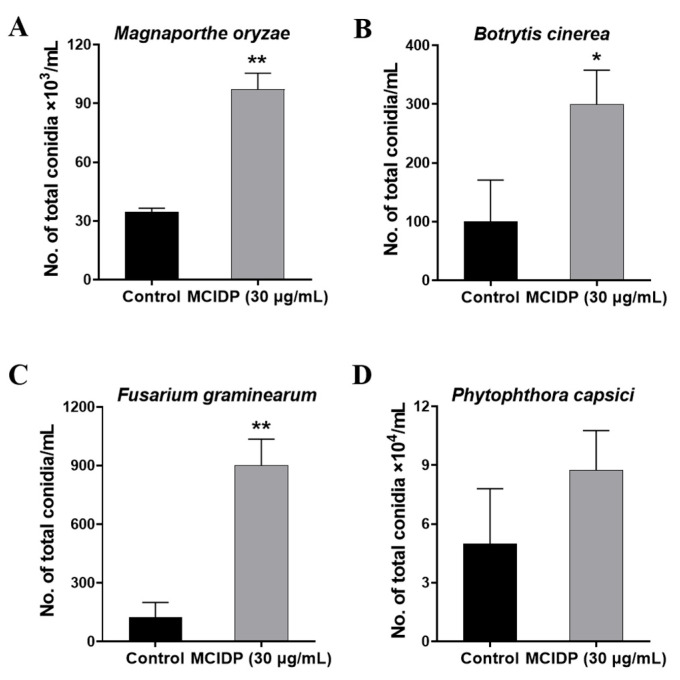
The effect of MCIDP on conidial formation of different fungi. Effects of 30 μg/mL MCIDP on the production of conidia in the *M. oryzae* (**A**), *B. cinerea* (**B**), *F. graminearum* (**C**), and *P. capsici* (**D**) strains were tested on agar plates; the negative control is sterilized water. *M. oryzae* was tested on CM plates. *B. cinerea* and *P. capsici* were tested on V8 juice agar plates, and *F. graminearum* was tested on PDA plates. The experiment was repeated three times. The data were expressed as a mean ± SEM. * and ** represent a significant difference as compared to the negative group at *p* < 0.05 and *p* < 0.01.

*M. oryzae*, *Botrytis cinerea (B. cinerea)*, *F*. *graminearum,* and *Phytophthora capsici (P. capsici)* were treated with 30 μg/mL MCIDP to explore the universality of promotion by MCIDP. Figure 3A displayed that MCIDP at 30 μg/mL significantly increased the number of conidia from (34 ± 3) × 10^3^ conidia/mL to (97 ± 14) × 10^3^ conidia/mL at 30 μg/mL. *B. cinerea* and *F. graminearum* displayed a more intimate phylogenetic relationship with *M. oryzae* compared to *P. capsici.* Both *B. cinerea* and *F. graminearum* are filamentous ascomycetes, and their crop-infection processes typically initiate from conidia [26,27]. *P*. *capsici* belongs to the *Oomycota* (flagellated organisms), whose first step of its plant-invasion process is sporangium production [28]. In the tested *B. cinerea* and *F. graminearum*, MCIDP also induced conidia formation but did not significantly induce sporangium formation in the tested *P. capsici* (Figure 3B–D). This finding suggested that MCIDP may have similar conidial formation-promoting activity in the two other filamentous ascomycete fungi.

### 2.4. MCIDP Upregulates the Genes Involved in the Mps1 MAPK Signaling Pathway

**Figure 4:**
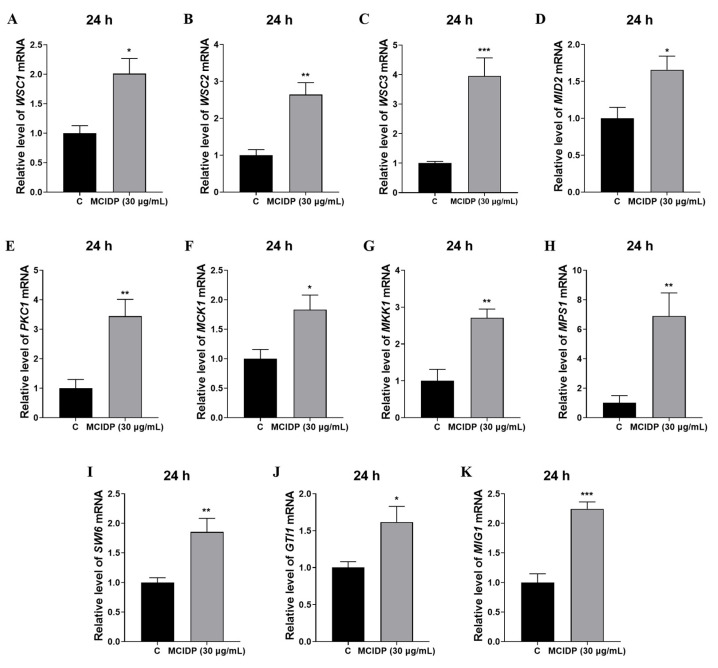
MCIDP elevated the expression of genes associated with the MAPK signaling pathway. (**A**–**D**) The expression of transmembrane protein genes *WSCs* and *MID2* after MCIDP treatment. (**E**–**H**) The expression of downstream core genes after treatment with MCIDP. (**I**–**K**) The expression of the main transcription factor genes is regulated by the Mps1 MAPK cascade after treatment with MCIDP. The experiment was repeated three times. The data were expressed as a mean ± SEM. *, **, and *** represent a significant difference compared to the control group at *p* < 0.05, *p* < 0.01, and *p* < 0.001.

RT-PCR analysis was applied to explore the effect of MCIDP on the genes involved in the Mps1 MAPK cascade pathway, which is crucial for promoting conidial formation. The core genes in the Mps1 MAPK signaling pathway, such as *MCK1*, *MKK1*, and *MPS1*, are highly conserved in fungi. Mck1-Mkk1-Mps1 cascades play critical roles in the conidial formation of *M. oryzae* [2]. The main transcription factors regulated by the Mps1 MAPK cascade include Mig1, Swi6 and Gti1 [16]. This study found that MCIDP exerted the most significant promoting effect on the gene expression of *WSC3* (Figure 4A–D). For the downstream core genes in this pathway, MCIDP could significantly upregulate the abundance of *PKC1*, *MCK1*, *MKK1*, and *MPS1*, and especially *MPS1* (Figure 4E–H). The main transcription factors regulated by the Mps1 MAPK cascade pathway were also upregulated (Figure 4I–K). These results showed that MCIDP upregulates the gene expression in the Mps1 MAPK cascade signaling pathway to promote the conidiation of *M. oryzae.*

### 2.5. Conidial Production Decreases in ΔMomid2 and ΔMowsc1 Mutants

**Figure 5:**
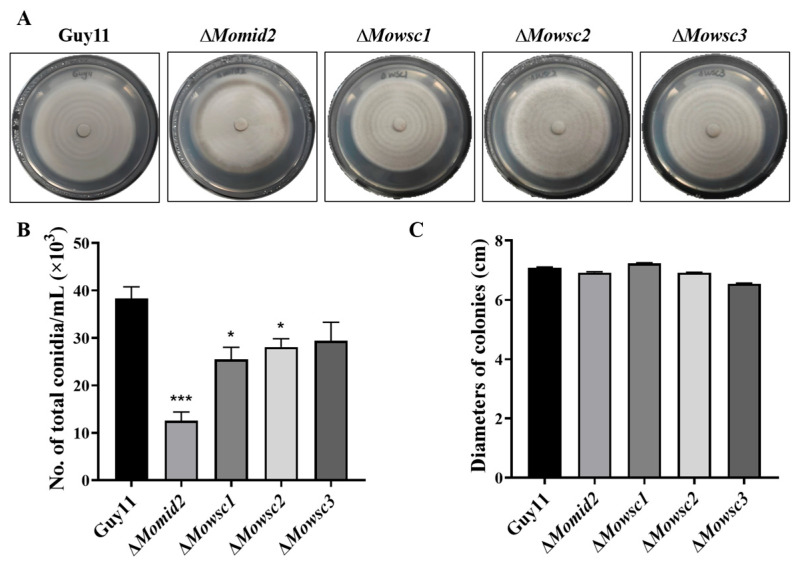
The conidiation and mycelial growth of *M. oryzae* wild-type strain Guy11, Δ*Momid2*, Δ*Mowsc1*, Δ*Mowsc2*, and Δ*Mowsc3* mutants. Colony morphology (**A**), the number of conidia analysis (**B**), and colony diameter analysis (**C**) of *M. oryzae* wild-type strain Guy11, Δ*Momid2*, Δ*Mowsc1*, Δ*Mowsc2*, and Δ*Mowsc3* mutants. Each plate was cultured at 25 °C for 10 days in the dark. The experiment was repeated three times. The data were expressed as a mean ± SEM. * and *** represent a significant difference compared to the control group at *p* < 0.05 and *p* < 0.001, respectively.

To confirm the importance of the Mps1 MAPK signaling pathway in the conidiation promotion of MCIDP, we constructed Δ*Mowsc1*, Δ*Mowsc2*, Δ*Mowsc3,* and Δ*Momid2* mutants using homologous recombination to replace the upstream sensors *Wsc1/2/3* and *Mid2* with the hygromycin gene. Dean et al. reported that the MAPK, cAMP, and Ca^2+^ signaling pathways are involved in regulating the conidiation of *M. oryzae* [2]. Among the reported MAPK signaling pathways, the Mps1 MAPK cascade pathway is activated by the membrane proteins Wscs (Wsc1/2/3) and Mid2 through Pkc1 and is crucial for conidial formation [16]. Figure S1 shows the results verified by PCR and agarose gel electrophoresis, and Figure 5A displays the colony morphology of these strains. The conidia of the Δ*Momid2* and Δ*Mowsc1* strains were significantly reduced compared with those of the parent strain Guy11 (Figure 5B). Meanwhile, no significant change in mycelium growth rate was observed between the wild-type strain Guy11 and all the mutants (Figure 5C).

### 2.6. The Membrane Proteins, Wscs and Mid2, in the Mps1 MAPK Signaling Pathway Are Critical for MCIDP to Promote Conidiation and Hyphal Growth

**Figure 6:**
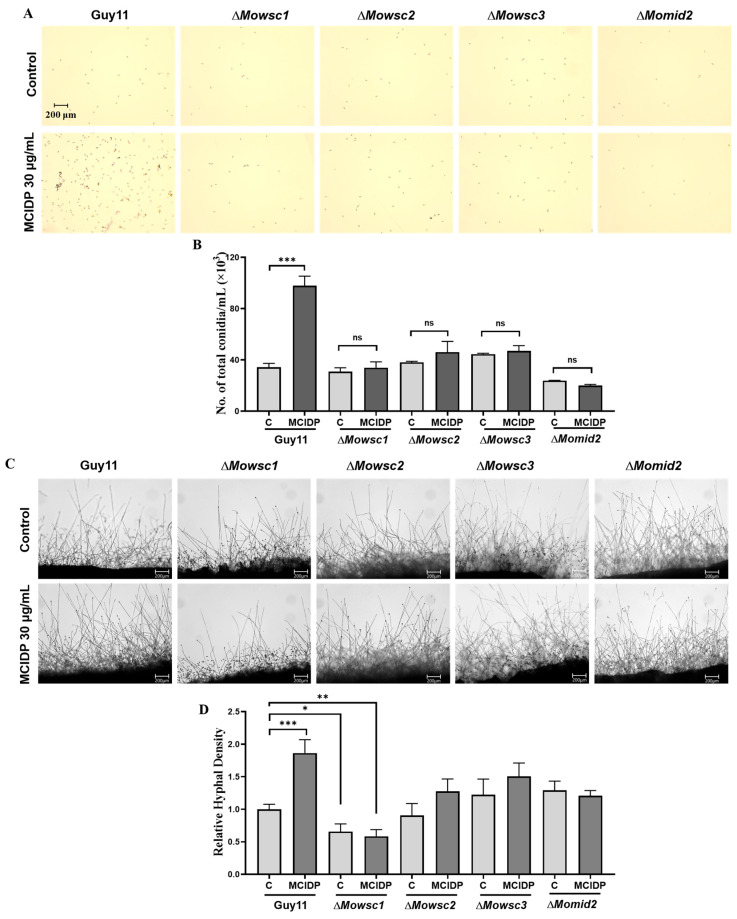
*WSCs* and *MID2* contribute to the efficacy of MCIDP in enhancing conidiation and hyphal growth. Micrograph of the conidia (**A**), the number of conidia (**B**), the aerial hyphae morphology (**C**), the relative hyphal density (**D**) of wild-type strain Guy11, the Δ*Mowsc1*, Δ*Mowsc2*, Δ*Mowsc3,* and Δ*Momid2* mutant with or without 30 μg/mL MCIDP treatment on CM agar plates. Scale bar: 200 μm. Each group was cultured at 25 °C for 10 days in the dark. The experiment was repeated three times. The data were expressed as a mean ± SEM. *, **, and *** represent a significant difference compared to the control group in the wild-type strain Guy11 at *p* < 0.05, *p* < 0.01, and *p* < 0.001. “ns” represents no significant difference compared to the control group.

After successfully constructing Δ*Mowsc1*, Δ*Mowsc2*, Δ*Mowsc3,* and Δ*Momid2* mutants from the *M. oryzae* wild-type strain Guy11, we further evaluated the changes in conidial formation and hyphal growth of these strains were further evaluated upon treatment with 30 μg/mL MCIDP (Figure 6). As shown in Figure 6A,B, MCIDP significantly increased the number of conidia from (34 ± 2) × 10^3^ to (97 ± 7) × 10^3^ in the wild-type strain Guy11. However, in the Δ*Mowsc1*, Δ*Mowsc2*, Δ*Mowsc3*, and Δ*Momid2* mutants, MCIDP failed to exert its conidial formation-promoting effect.

Observation of aerial hyphae under a microscope revealed that MCIDP could promote spore germination and increase hyphal density in the wild-type strain Guy11 (Figure 6C,D). In the Δ*Mowsc1* strain, the hyphal density was significantly reduced compared with that in the parent strain Guy11. Meanwhile, there was no significant change in mycelium growth status observed between the Δ*Mowsc2*, Δ*Mowsc3*, and Δ*Momid2* mutants and the wild-type strain Guy11. Additionally, MCIDP had no effect on hyphal growth in the Δ*Mowsc1*, Δ*Mowsc2*, Δ*Mowsc3*, and Δ*Momid2* strains (Figure 6C,D). These data indicated that the *WSC1*, *WSC2*, *WSC3*, and *MID2* genes in the Mps1 MAPK signaling pathway are critical for MCIDP to promote conidiation.

## 3. Discussion

In this study, a decapeptide (MCIDP) isolated from AHC could significantly and dose-dependently promote the conidiation of the phytopathogenic fungus *M. oryzae* in the dose range of 30 ng/mL to 30 μg/mL (Figure 1 and Figure 2). The potential for MCIDP to promote conidiation was further examined across several other plant pathogens, namely, *B. cinerea*, *F. graminearum,* and *P. capsici*. Our findings suggested that MCIDP possessed analogous conidial formation activity in the two other measured filamentous ascomycete fungi, *B. cinerea* and *F. graminearum*, but not in the oomycete phytopathogenic *Phytophthora* (Figure 3). To elucidate the mechanism of MCIDP inducing the conidial formation in *M. oryzae*, we evaluated the expression of the genes involved in the Mps1 MAPK signaling pathway, which is crucial for conidiation, and constructed relevant mutants. The results showed that MCIDP could significantly upregulate the pivotal genes of the Mps1 MAPK signaling pathway, especially *WSC2*, *WSC3*, *PKC1*, *MKK1*, *MPS1*, and *MIG1.* Additionally, MCIDP did not increase the conidiation in Δ*Mowsc1*, Δ*Mowsc2*, Δ*Mowsc3*, and Δ*Momid2* mutants (Figure 4, Figure 5 and Figure 6). These results indicated that the conidiation-promoting activity of MCIDP is closely related to the Mps1 MAPK signaling pathway.

As an inducer of asexual reproduction, MCIDP could induce conidiation in two other filamentous ascomycetes: *B. cinerea* and *F. graminearum,* which belong to different genera than *M. oryzae*. Our previous study found that FARI, a *Fusarium* asexual reproduction inducer, promotes conidial formation only in the *Fusarium* genus, but does not affect the conidia production of other filamentous fungi such as *B*. *cinerea* and *Penicillium digitatum* [12]. We speculate that this discrepancy might arise because FARI, as an endogenous signaling molecule produced by *F. graminearum*, is highly specific and causes conidiation-inducing activity only in the fungi of the *Fusarium* genus. Meanwhile, MCIDP, as an exogenous inducer, could function across different genera of *Ascomycota* fungi. Additionally, Qi et al. successfully isolated sexual reproductive hormones α1 and α2 from *Phytophthora nicotianae*, which induced oospore production on the mating types of *Phytophthora* A2 and A1 at a dose of 3 ng, respectively [9,10,11]. Endogenous signaling molecules have the characteristics of being trace, efficient, and highly specific. Finding the endogenous signaling molecules of asexual reproduction and their specific receptors is of great significance for the discovery of new specific antibiotics or antibacterial agents. Hence, we are actively searching for endogenous signaling molecules that promote conidiation in *M. oryzae*.

Our findings are in accordance with earlier studies that have underscored the pivotal role of the Mps1 MAPK signaling pathway in the asexual reproduction and conidia formation of *M. oryzae*. For instance, Dean et al. emphasized that the MAPK signaling pathways are essential for the growth, development, and conidial formation of *M. oryzae*, with the Mps1 MAPK cascade being particularly vital for conidial production [2]. The Mps1 MAPK cascades could promote the various stages in the asexual lifecycle of *M. oryzae* [15,16]. Consistent with these findings, our study revealed that MCIDP upregulates the key genes in the Mps1 MAPK pathway to promote conidiation. This discovery offers a novel perspective on how exogenous molecules can influence fungal asexual reproduction and provides important technical support and methodological basis for studying the function and molecular mechanism of endogenous signaling molecules.

Although this study has yielded significant insights, it has several limitations that warrant acknowledgment. We found that MCIDP promotes conidiation via the Mps1 MAPK signaling pathway, but its target of MCIDP remains to be elucidated. Future investigations should concentrate on delineating the intricate interactions between MCIDP and its target by employing techniques such as RNA sequencing, surface plasmon resonance (SPR), and co-immunoprecipitation (Co-IP). A more comprehensive understanding of the signaling cascade would necessitate exploring changes in protein expression and subcellular localization in response to MCIDP treatment.

On the basis of the findings of this study, several promising avenues for future research can be proposed. The development of inhibitors targeting the Mps1 MAPK signaling pathway could provide new strategies for controlling rice blast disease. Conidiation and pathogenicity in *M. oryzae* can be reduced by inhibiting this pathway, thereby protecting rice crops from infection. As previously reported by Rika I. et al., the compound lycosides A-D isolated from vegetable juices, a typical culture medium of *Phytophthora*, inhibits asexual reproduction of the plant pathogen *Phytophthora* [14]. Hence, further explorations of the bioactive molecules in AHC and other compositions of the CM medium could lead to the identification of additional regulators of conidia formation.

**Figure 7:**
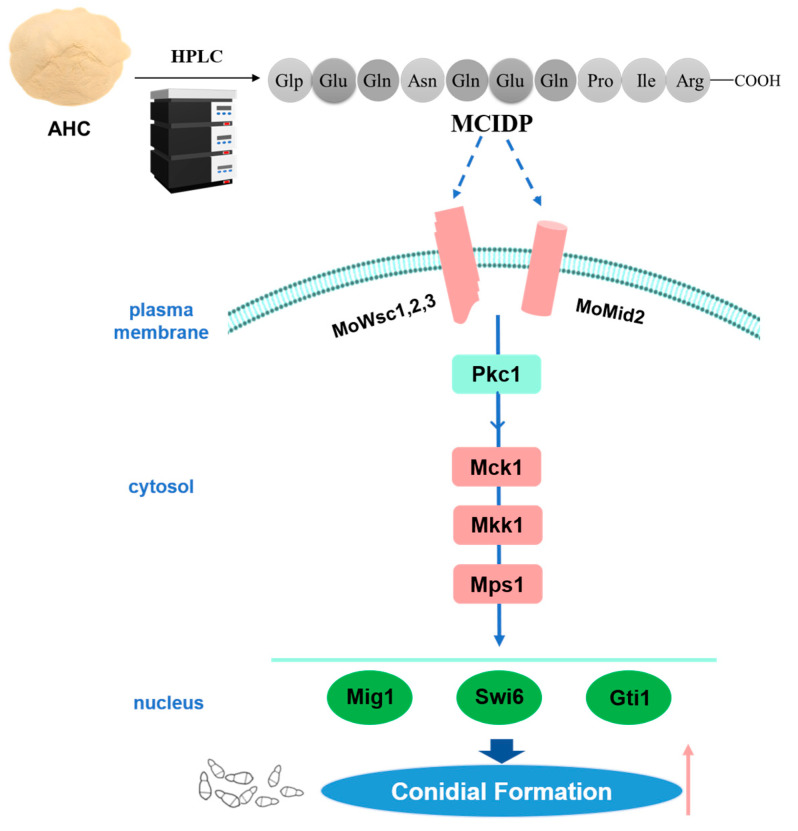
Proposed potential mechanism of action for MCIDP. MCIDP promotes conidial formation by increasing the gene expression in the Mps1 MAPK cascade signaling pathway.

In conclusion, this study provides valuable insights into the role of MCIDP in promoting conidiation in *M. oryzae* through the Mps1 MAPK signaling pathway (Figure 7). Despite the limitations of our current findings, this study establishes a technical foundation for the subsequent exploration of fungal endogenous signaling molecules and provides novel perspectives for developing new strategies to control rice blast and other fungal infections.

## 4. Materials and Methods

### 4.1. General

Analytical pure reagents (chloroform, isopropanol, and ethanol from Sinopharm Chemical Reagent Co., Ltd., Shanghai, China) and chromatography-grade acetonitrile (TEDIA, Toledo, OH, USA) were used for the isolation and purification. Preparative HPLC analysis was conducted using an HPLC system equipped with Elite P1100 pumps and a D1100 UV detector (Dalian Elite Inc., Dalian, China). High-resolution electrospray ionization mass spectrometry (HR-MS) analysis was performed on an Agilent 6224A accurate mass time-of-flight LC/MS system (Agilent Technologies Inc., Beijing, China). CFW (0.5 g/L, Sigma-Aldrich, Saint Louis, MO, USA) was used for conidia staining. The following reagents were purchased from designated suppliers for the preparation of required culture media: various metal salts and sucrose (Sinopharm Chemical Reagent Co., Ltd., Shanghai, China), D-(+)-Glucose, agar and acid-hydrolyzed casein (Sigma-Aldrich, Saint Louis, MO, USA), yeast extract, tryptone, and peptone (Oxoid, Basingstoke, UK), required vitamins (Aladdin Biochemical Technology Co., Ltd., Shanghai, China), and V8 juice (Campbell Soup, Camden, NJ, USA).

### 4.2. Strains and Culture Conditions

*M. oryzae* strain Guy11 was used as the wild-type strain. All strains were cultured on solid CM for growth at 25 °C with a 12 h light-dark cycle. The compositions of the CM medium were provided in Supplementary Tables S1–S4. The previous report can be referred to for specific cultural conditions [13].

### 4.3. Preparation of MCIDP from AHC

AHC was purchased from Sigma-Aldrich Co., Ltd. AHC (24 mg) was purified first through HPLC (NH2P-50 10E (ϕ 10 × 250 mm)) using 100%–20% aqueous acetonitrile gradient elution for 30 min, 20% aqueous acetonitrile elution for 20 min, the flow rate of 3 mL/min and the detection wavelength of 210 nm to obtain an active fraction (2.7 mg, t_R_ = 16.8 min). Then, the active fraction was further purified by HPLC (NH2P-50 10E (ϕ 10 × 250 mm)) using 85% aqueous acetonitrile elution for 30 min, the flow rate of 3 mL/min and the detection wavelength of 210 nm to obtain the active sample (2.4 mg, t_R_ = 12.3 min). To accurately identify the amino acid sequence of the active sample, it was entrusted to Bioengineering (Shanghai) Co., Ltd. (Shanghai, China) for peptide sequencing using LC-MS/MS technology. HR ESI-TOF-MS *m/z* 1252.5918 and 626.7998, which were calculated for C_51_H_82_N_17_O_20_^+^ [M + H]^+^ 1252.5917 and C_51_H_83_N_17_O_20_^2+^ [M + 2H]^2+^ 626.7995, respectively. The identification results showed that the amino acid sequence was as follows: pryoGlu-EQNQEQPIR, the sequence was reported in the literature [29]. This compound was designated as MCIDP, which was used in subsequent experiments, and was synthesized by Hangzhou Specialized Peptide Biotechnology Co., Ltd. (Hangzhou, China). The sequence of MCIDP was shown in Figure 2A.

### 4.4. Conidium Collection and CFW Staining

*M. oryzae* strains were cultured for 6–8 days at 25 °C. Sterilized water (5 mL) was added to each plate, and mycelia were scraped off and rinsed to collect conidia. The collected liquid was filtered, and the filtrate was centrifuged at 4000 rpm for 10 min. After discarding the supernatant, the pellet was resuspended and counted. The ratio of conidial suspension to CFW (Calcofluor White) dye solution 10:1 was used to stain the conidium for 3 min, and the conidium was viewed under the fluorescence microscope (Olympus BX61, Tokyo, Japan).

### 4.5. 24-Well Plate Liquid Culture Bioassay Method

Then, 1 mL of 0.3 × CM liquid medium and 10^4^ conidia were added to one well of a 24-well plate. The plate was incubated at 25 °C in the dark for 36 h to form a mycelial mat. Samples were added beneath the mat and cultured for an additional 24 h. After vigorous mixing, conidia were counted under an optical microscope (Olympus, Tokyo, Japan) [2,30].

### 4.6. Quantitative Real-Time Polymerase Chain Reaction (qRT-PCR) Analysis

Here, 3 × 3 cm fungal colonies were cut and triturated, then cultured in 300 mL CM liquid medium at 28 °C, 180 rpm for 40 h. Mycelia were harvested, and large hyphal fragments were removed, then resuspended in an equal volume of 0.3 × CM medium. Then, 10 mL mycelial suspension was transferred to a dish with a diameter of 6 cm and incubated at 25 °C with 12 h light/dark cycles for 2 days until surface pellicle formation. After this, 30 μg/mL MCIDP was added under the pellicle for 24 h treatment, then the hyphal-interwoven pellicle was collected. RNA from mycelium was extracted using the TRIzon Reagent (CW0580S, CoWin Biotech, Beijing, China). The cDNA samples were prepared using HiScript III QRT SuperMix for qPCR (+gDNA wiper) (Vazyme Biotech, Nanjing, China). Primers used in quantitative RT-PCR are displayed in Table S5 RT-PCR was conducted on a Bio-Rad CFX96 real-time system (Bio-Rad, Hercules CA, USA) with SYBR Premix EX Taq™ (Takara, Otsu, Japan). All results were standardized to actin levels, and the relative mRNA transcript levels were calculated by the 2^−∆∆Ct^ method.

### 4.7. Generation of MoWsc1, MoWsc2, MoWsc3 and MoMid2 Gene Deletion Mutants

The genome sequences of MoWsc1 (MGG_04325), MoWsc2 (MGG_09412), MoWsc3 (MGG_00066) and MoMid2 (MGG_12606) were obtained from the website EnsemblFungi https://fungi.ensembl.org/Multi/Search/New?db=core (accessed on 30 May 2024). Based on homologous recombination technology, primer pairs named UP-F/R and DOWN-F/R (Table S6) were used to amplify the 1.5 kb sequences upstream and downstream of the target gene, respectively. Subsequently, these PCR products were ligated with the hygromycin B phosphotransferase (HPH) gene cassette and cloned into the EcoRI and XhoI restriction sites of the pKOV21 vector, thereby generating the gene knockout vector (Figure S1A). Then, transform the obtained vector into protoplasts of the Guy11 strain. The mutants were selected on a CM medium supplemented with a final concentration of 300 µg/mL HPH and further confirmed by PCR amplification with the primer pairs YW-F/R (gene internal primers) (Table S7). The transformants that cannot be amplified by YW-F/R were selected and further verified for the length of the inserted fragments using DX-F/R (gene external primers) (Table S7). The agarose gel electrophoresis verification results of Δ*Mowsc1*, Δ*Mowsc2*, Δ*Mowsc3*, and Δ*Momid2* mutant strains are shown in Figure S1B–M.

### 4.8. Phenotypic Analysis

#### 4.8.1. Assessment of Mycelial Growth

Mycelial growth was evaluated by measuring colony diameters on CM plates after incubation at 25 °C for 10 days.

#### 4.8.2. Conidial Counting

A hole punch was used to randomly remove a sample 0.6 cm in diameter from the fungus. It was put into an Eppendorf (EP) tube containing 1 mL of water for counting after vortexing. The experiment was repeated three times.

#### 4.8.3. Conidial Imaging

Conidia, after 10 days of cultivation on CM plates supplemented with samples, were collected, centrifuged at 5000 rpm for 10 min, and the supernatant was discarded. The volume of each sample was fixed to 500 μL, and the sample was vortex-mixed and then photographed under the microscope (Olympus, Tokyo, Japan).

#### 4.8.4. Lateral Imaging of Conidiophore and Hyphae

For observing the formation of aerial hyphae and conidiation, 1 mm-thick sections of each fungal strain’s colony were carefully excised and placed on a slide. These slides were then incubated at 28 °C for 24 h under moisturizing conditions and then photographed under the microscope (Olympus, Tokyo, Japan).

### 4.9. Data Processing and Analysis

Experiments were repeated three times, and the data were presented as mean ± SEM. The data were analyzed via *t*-tests and one-way ANOVA, followed by Tukey’s Multiple Comparison Test on GraphPad Prism software (Version 9.0.0 (121), GraphPad Prism, San Diego, CA, USA).

## Data Availability

The data supporting the conclusions of this study can be acquired from the corresponding author upon a reasonable request.

## Supplementary Materials

The following supporting information can be downloaded at: https://www.mdpi.com/article/10.3390/ijms26125880/s1.

## Abbreviations

The following abbreviations are used in this manuscript:

*M. oryzae*	*Magnaporthe oryzae*
CM	Complete Medium
AHC	Acid Hydrolyzed Casein
HPLC	High-Performance Liquid Chromatography
MCIDP	*M. oryzae* Conidiation Inducer Decapeptide
FARI	*Fusarium* Asexual Reproduction Inducer
*F. graminearum*	*Fusarium**g**raminearum*
MAPK	Mitogen-Activated Protein Kinase
CFW	Calcofluor White
*B. cinerea*	*Botrytis cinerea*
*P. capsic**i*	*Phytophthora capsic**i*
