# Supplementary material for: Decapeptide Inducer Promotes the Conidiation of Phytopathogenic Magnaporthe oryzae via the Mps1 MAPK Signaling Pathway
Source: Int J Mol Sci. 2025 Jun 19;26(12):5880. doi: 10.3390/ijms26125880 (PMC12192705; doi:10.3390/ijms26125880)
Supplement: Supplementary file 1 [file ijms-26-05880-s001.zip › ijms-3647489-supplementary.pdf]

Supplementary information

Decapeptide Inducer Promotes the Conidiation of Phytopathogenic *Magnaporthe oryzae* via the Mps1 MAPK Signaling Pathway

Mengya Yang †, Yanan Liu † and Jianhua Qi *

College of Pharmaceutical Science, Zhejiang University, Yu Hang Tang Road 866, Hangzhou 310058, China; 22219083@zju.edu.cn (M.Y.); liuyanan1231@zju.edu.cn (Y.L.)

* Correspondence: qijianhua@zju.edu.cn; Tel.: +86-0571-88208627

† These authors contributed equally to this work.

Supplementary Tables

Supplementary Table S1. The component of complete medium (CM)

Component	Dosage
1000× Trace elements solution	1 mL
1000× Vitamin solution	1 mL
Acid-hydrolyzed casein	1 g
Yeast extract	1 g
Peptone	2 g
D-glucose	10 g
20× Nitrate salts	50 mL
0.2 M NaOH	15 mL
Agar	15 g
H_2O	Add to 1 L

* Prepare liquid CM medium by omitting agar from the aforementioned recipe.

* 0.3× CM liquid medium is prepared by adding each component of the aforementioned recipe at 0.3 times the original proportions.

Supplementary Table S2. The component of 1000× Vitamin solution

Component	Dosage
p-Aminobenzoic acid	0.01 g
Nicotinic acid	0.01 g
Vitamin B2	0.01 g
Vitamin B6	0.01 g
Vitamin B1	0.01 g
Vitamin H	0.01 g
H_2O	Add to 100 mL

Supplementary Table S3. The component of 1000× Trace elements solution

Component	Dosage
$ZnSO_4{\cdot}7H_2O$	2.20 g
H_3BO_3	1.10 g
$MgCl_2{\cdot}4H_2O$	0.50 g
$FeSO_4{\cdot}7H_2O$	0.50 g
$CoCl_2{\cdot}6H_2O$	0.17 g
$CuSO_4{\cdot}5H_2O$	0.16 g
$Na_2MoO_4{\cdot}5H_2O$	0.15 g
Na_4EDTA	5.00 g
H_2O	Add to 100 mL

Supplementary Table S4. The component of 20× Nitrate salts

Component	Dosage
$NaNO_3$	120.0 g
KH_2PO_4	30.4 g
KCl	10.4 g
$MgSO_4 \cdot 7H_2O$	10.4 g
H_2O	Add to 1 L

Supplementary Table S5. The sequences of the primers for RT-PCR analysis

Primers	Sequences	Gene ID
Wsc1, sense	5′- TACTGGTGCCTCGTGCTAC -3′	MGG_04325
antisense	5′- TGCTTGCCTGTGTTATAGATGG -3′	
Wsc2, sense	5′- TTACTCACCTGCCGCTCAC -3′	MGG_09412
antisense	5′- GGATAGTTCCACTGGCTGTAG -3′	
Wsc3, sense	5′- AATCGTCGGTGGCAACTCT -3′	MGG_00066
antisense	5′- ATCTGCTGCTGCTGACCA -3′	
Mid2, sense	5′- GCATCTATCGCTGGTCCTCA -3′	MGG_12606
antisense	5′- ACAGTGGTTATGGTGGTTCTTG -3′	
Mck1, sense	5′- CGAGACTGTTCTTGACCTGAG-3′	MGG_00883
antisense	5′- GCGTGCCTCTTCTGATTGAT-3′	
Mkk1, sense	5′- CCATCTACCGCAACAATCTCTAT-3′	MGG_06482
antisense	5′- TCCGATGTTATGGTGTAACTCTG-3′	
Mps1, sense	5′- TTGATGTATGGTCGGTTGGATG -3′	MGG_04943
antisense	5′- ATCGTGCCAGATGTGGAGATA -3′	
Pkc1, sense	5′- CAGAGGTGAAGACGAAGATGAG -3′	MGG_08689
antisense	5′- CAGACGATGTTGCGGAAGAA -3′	
Mig1, sense	5′- GGCGATGACGATGATGAGGA -3′	MGG_01204
antisense	5′- GGCGAGGCTGAAGGTGTATA -3′	
Swi6, sense	5′- ACCACAGCAGCCACAAGAA -3′	MGG_09869
antisense	5′- CATAACAGCAACGCCATTGAC -3′	
Gti1, sense	5′- TGAACAGCAACGGCAACAA -3′	MGG_08850
antisense	5′- GGTCGGTGGATGGTGTAGT -3′	
Actin, sense	5′- GGTCTTGAGAGCGGTGGTAT -3′	MGG_03982
antisense	5′- ACTTGCGGTGGACAATGGA -3′	

Supplementary Table S6. The sequences of the primers for up/down stream amplification

Primers	Sequences
WSC1-UP-F	5′- CCCCCGGGCTGCAGGAATTC GTTCATCCTGGGACGCTTTG -3′
WSC1-UP-R	5′- GCTCCTTCAATATCATCTTCTCTCG GCAGACTGGATCAGGAGTTCT -3′
WSC1-DOWN-F	5′- TAGAGTAGATGCCGACCGAACAAGA GCCAAGGTGGTTGTTGTAGTTA -3′
WSC1-DOWN-R	5′- TACCGGGCCCCCCCTCGAG CCAGCAGGGACGTTGTCTT -3′
WSC2-UP-F	5'- CCCCCGGGCTGCAGGAATTCTTGGTGATCTTGGTGAGC -3′
WSC2-UP-R	5'- GCTCCTTCAATATCATCTTCTCTCGATCTGGCTTGAAGGTGAG -3′
WSC2-DOWN-F	5′- TAGAGTAGATGCCGACCGAACAAGAACTCTCGTCTGCTTGAAG -3′
WSC2-DOWN-R	5′- TACCGGGCCCCCCCTCGAGCGCTATGGTGTGAACTCT -3′
WSC3-UP-F	5′- CCCCCGGGCTGCAGGAATTCATCACAACGACGGCGAAAT -3′
WSC3-UP-R	5′- GCTCCTTCAATATCATCTTCTCTCGCCAACAGCAGCAACAATAGG 3′
WSC3-DOWN-F	5′- TAGAGTAGATGCCGACCGAACAAGAGACTGAAGGAGTTCGT -3′
WSC3-DOWN-R	5′- TACCGGGCCCCCCCTCGAGCGGATGTGCAATGAGT -3′
MID2-UP-F	5′- CCCCCGGGCTGCAGGAATTC CGCTGACTTCACACCTCATAG -3′
MID2-UP-R	5′- GCTCCTTCAATATCATCTTCTCTCG ACCCAGAGGCGTTGCTTT -3′
MID2-DOWN-F	5′- TAGAGTAGATGCCGACCGAACAAGA CGTCACAGTTTACGGCTTT -3′
MID2-DOWN-R	5′- TACCGGGCCCCCCCTCGAG CGTGTCGAACTTTGGAGGA -3′

Supplementary Table S7. The sequences of the primers for mutant verification

Primers	Sequences
WSC1-YW-F	5′-AGCAACACGGCAGGCATT-3′
WSC1-YW-R	5′-ACACCTTCACACTGACGAGAG-3′
WSC1-DX-F	5′-ATGCAACTAGGTAGGTCCGT-3′
WSC1-DX-R	5′-GGACGCTGATGGAACTGGT-3′
WSC2-YW-F	5'- GTAGGCAAGGTAGCAGATGATT -3′
WSC2-YW-R	5'-TGAACGAGGACGAAGAGACA-3′
WSC2-DX-F	5′- GTAGCCACCGACAAGGAT -3′
WSC2-DX-R	5′- CCAACAAGGAGATGAAGCA -3′
WSC3-YW-F	5′- CGCCTATTGTTGCTGCTGTT -3′
WSC3-YW-R	5′- ATCTGCTGCTGCTGACCAC -3′
WSC3-DX-F	5′- GCTGTGATACGCACTATG -3′
WSC3-DX-R	5′- GCCTACCTACCTACCTAAG -3′
MID2-YW-F	5′-GTTATCACTGCTGTGGCT-3′
MID2-YW-R	5′-CCATCGCTATTGGTTGTAG-3′
MID2-DX-F	5′-CCGTCTGCATCTTTTGAGCT-3′
MID2-DX-R	5′-GGTAGATGACGCCCATTCT-3′
Hph-DX-F*	5′- AAGCAAGGTAAGTGAACGA-3′
Hph-DX-R*	5′- GCCTCCAGAAGAAGATGTT-3′
Hph-F#	5′- CGAGAGAAGATGATATTGAAGGAGC-3′
Hph-R#	5′- CGAGAGAAGATGATATTGAAGGAGC-3′

* Validation primer pair was designed on the inside of the hygromycin resistance gene fragment.

# Primer pair was designed to amplify the hygromycin gene fragment.

Supplementary Figures

Supplementary Figure S1.

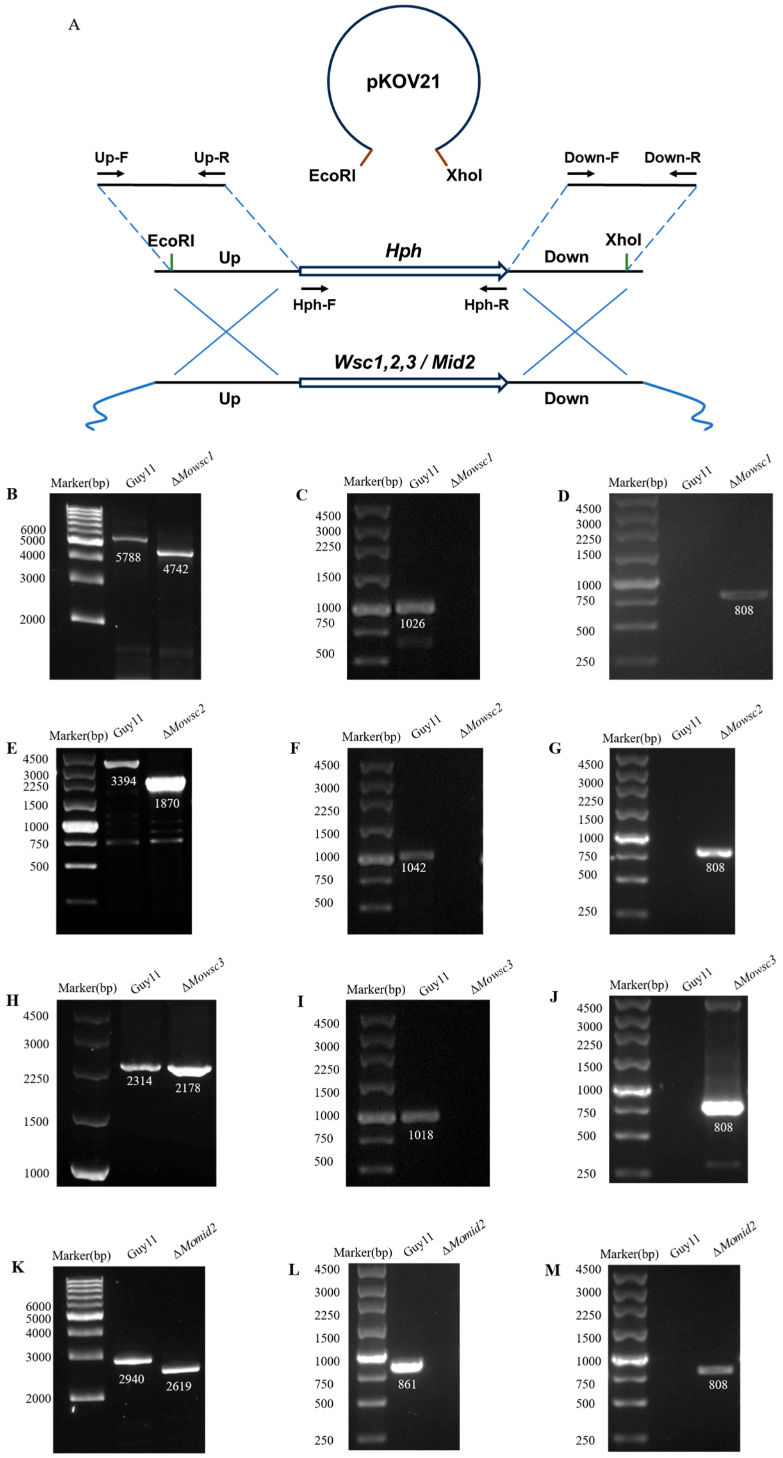

Figure S1. Schematic diagram of gene knockout principle and agarose gel electrophoresis verification results for *Magnaporthe oryzae* mutants. (A) Construction of the knockout vector pKO-Wsc1/Wsc2/Wsc3/Mid2 and gene replacement. (B-M) PCR validation of Wsc1 (B-D), Wsc2 (E-G), Wsc3 (H-J), and Mid2 (K-M) gene knockout results. Primer pairs were designed on the outside (B, E, H, and K) and inside (C, F, I, and L) of the target knockout gene WSC1 fragment and the inside of the resistance gene fragment (D, G, J, and M), and the results were verified by PCR and agarose gel electrophoresis.
